# Implications on the Therapeutic Potential of Statins *via* Modulation of Autophagy

**DOI:** 10.1155/2021/9599608

**Published:** 2021-07-30

**Authors:** Armita Mahdavi Gorabi, Nasim Kiaie, Saeed Aslani, Thozhukat Sathyapalan, Tannaz Jamialahmadi, Amirhossein Sahebkar

**Affiliations:** ^1^Research Center for Advanced Technologies in Cardiovascular Medicine, Tehran Heart Center, Tehran University of Medical Sciences, Tehran, Iran; ^2^Department of Immunology, School of Medicine, Tehran University of Medical Sciences, Tehran, Iran; ^3^Department of Academic Diabetes, Endocrinology and Metabolism, Hull York Medical School, University of Hull, Hull, UK; ^4^Department of Food Science and Technology, Quchan Branch, Islamic Azad University, Quchan, Iran; ^5^Department of Nutrition, Faculty of Medicine, Mashhad University of Medical Sciences, Mashhad, Iran; ^6^Applied Biomedical Research Center, Mashhad University of Medical Sciences, Mashhad, Iran; ^7^Biotechnology Research Center, Pharmaceutical Technology Institute, Mashhad University of Medical Sciences, Mashhad, Iran; ^8^School of Pharmacy, Mashhad University of Medical Sciences, Mashhad, Iran

## Abstract

Statins, which are functionally known as 3-hydroxy-3-methyl-glutaryl-CoA (HMG-CoA) inhibitors, are lipid-lowering compounds widely prescribed in patients with cardiovascular diseases (CVD). Several biological and therapeutic functions have been attributed to statins, including neuroprotection, antioxidation, anti-inflammation, and anticancer effects. Pharmacological characteristics of statins have been attributed to their involvement in the modulation of several cellular signaling pathways. Over the past few years, the therapeutic role of statins has partially been attributed to the induction of autophagy, which is critical in maintaining cellular homeostasis and accounts for the removal of unfavorable cells or specific organelles within cells. Dysregulated mechanisms of the autophagy pathway have been attributed to the etiopathogenesis of various disorders, including neurodegenerative disorders, malignancies, infections, and even aging. Autophagy functions as a double-edged sword during tumor metastasis. On the one hand, it plays a role in inhibiting metastasis through restricting necrosis of tumor cells, suppressing the infiltration of the inflammatory cell to the tumor niche, and generating the release of mediators that induce potent immune responses against tumor cells. On the other hand, autophagy has also been associated with promoting tumor metastasis. Several anticancer medications which are aimed at inducing autophagy in the tumor cells are related to statins. This review article discusses the implications of statins in the induction of autophagy and, hence, the treatment of various disorders.

## 1. Introduction

Autophagy is crucial for maintaining the homeostasis of cells, both in physiological and pathological conditions [1, 2]. In the normal state, autophagy is involved in the degradation and clearance of the nonfunctional or aged cells and cell organelles that are potentially hazardous for cell survival [3, 4]. Cells need to maintain the balance between cell death and survival to modulate normal physiology and maintain homeostasis [5]. Nonetheless, if the cell is provided with limited amounts of nutrients, autophagy of the unnecessary organelles confers a limitation of energy demands, ensuring cell survival [6]. Dysregulated autophagy in molecular levels has been associated with the etiology and pathogenesis of various disorders, including autoimmunity [7], malignancy [8], and neurodegenerative diseases [9, 10]. Autophagy functions as a double-edged sword during tumor metastasis. On the one hand, it plays a role in inhibiting metastasis through restricting necrosis of tumor cells, suppressing the infiltration of the inflammatory cell to tumor niche, and developing the release of mediators that induce potent immune responses against tumor cells. On the other hand, autophagy has also been associated with promoting tumor metastasis [11]. Studies have revealed that autophagy promotes drug resistance in ovarian cancer cells, leading to tumor cell survival [12, 13]. On the contrary, inhibition of apoptosis has been associated with increased toxicity of cancer drugs against tumor cells [14]. In addition to cancer, the impairment of autophagy interferes with the clearance of amyloid-beta, leading to the development of Alzheimer's disease (AD) [15]. Consequently, the modulation of autophagy has been on track recently as a therapeutic strategy in treating neurodegenerative disorders [16].

Statins pharmacologically are inhibitors of the 3-hydroxy-3-methyl-glutaryl-CoA (HMG-CoA) reductase that primarily decrease low-density lipoprotein-cholesterol levels (LDL-C) and triglyceride. For a long time, statins have been prescribed for patients with higher levels of cholesterol, LDL-C, and hypertriglyceridemia in patients with cardiovascular disorders and diabetes [17, 18]. However, the current line of evidence has shown that statins have numerous lipid-independent (pleiotropic) actions [19–26]. Among the pleiotropic effects of statins is modulation of autophagy in various cells, providing a promising therapeutic strategy in treating disorders with impaired autophagy as primary underlying pathogenesis [27, 28].

In this review, we focus on the molecular pathways of autophagy and those modified by statins and try to discuss the implications of statins in the therapy of disorders related to the regulation of autophagy.

## 2. Autophagy in Depth

If autophagy mechanisms could not reduce stress levels and reverse the cell injury in the organelles, apoptosis-associated cell death occurs [29], called ferroptosis [30]. Several stress-related factors, such as a limited level of nutrients and cellular energy, increased rate of reactive oxygen species (ROS), and accumulation of aggregated and misfolded proteins, may trigger autophagy [31, 32]. Autophagy is manifested in three ways: microautophagy, macroautophagy, and chaperone-mediated autophagy [32, 33]. Macroautophagy, the primary form of autophagy, is responsible for the degradation of most proportion of the cytoplasmic cargos [34]. Autophagy-related genes (Atgs) are involved in the function of the macroautophagy, which is functionally involved in the degradation of cytoplasmic components in lysosomes, maturation of the phagosome, and exocytosis [35]. Microautophagy, considered the nonselective lysosomal degradative, is mediated through the direct surrounding of a cytoplasmic cargo via autophagic tubes. Microautophagy of soluble substrates in the cell is commonly stimulated by nitrogen starvation or rapamycin that activates signaling pathways. The significant microautophagy roles are maintaining the size of organelles, homeostasis of the cell membrane, and modulating the cell survival during nitrogen deprivation [36]. Like heat-shock proteins (HSPs), several chaperones are involved in recognising aggregated or misfolded proteins and directing them towards lysosomes for degradation or disaggregation [37]. In case chaperons could not resolve aggregated or misfolded proteins, autophagy-related cell death occurs [38].

After being discovered in 1963, autophagy signaling pathways have much been studied in the context of several human diseases [39, 40]. The four stages of autophagy include nucleation, elongation, maturation, and degradation [41]. At the first stage, translocation of the Unc-51 like autophagy activating kinase 1 (ULK1) initiation complex to the early autophagosome (or phagophore) occurs. Inhibition of the mammalian target of rapamycin (mTOR) leads to the release of the ULK1 complex, promoting the activation and translocation of the complex. In the second stage, the class III phosphatidyl triiodide (PI3) kinase complex facilitates the elongation of the phagophore membrane. Next, two autophagy receptors, including p62/sequestosome-1 and optineurin, promote the cargo's recruitment to the expanding phagophore. These two receptors are ligated with microtubule-associated protein light chain 3- (LC3-) II on the expanding phagophore through LC3-interacting regions (LIRs) and with polyubiquitin chains on autophagy substrates through the ubiquitin-like (Ubl) domains. At the third step, the maturation of the phagophore occurred by fusion of the phagophore with lysosome upon the recruitment of substrate and its enclosure. Finally, at the fourth step, the fusion of the autophagosome with lysosome results in the degradation of the autophagic substrates via acid hydrolases located in the lysosome. Degradation causes the recycling of nutrients inside the phagolysosomes to the cell cytosol [42].

The critical regulator of autophagy is mTOR [43, 44]. During the standard physiological settings with no stress and enough energy, mTOR signaling interrupts the ULK1 and PI3 kinase/vacuolar protein sorting protein 34 (Vps34) complex, resulting in the inhibition of autophagy. ULK1 and PI3 kinase/Vps34 complex are critical in composing the autophagosome structure [45]. During stress situations, mTOR signaling is modulated, resulting in activation of the ULK1 and PI3K/Vps43 complex. Beclin-1 is also involved in the development of autophagosomes [46, 47]. The adenosine monophosphate-activated protein kinase (AMPK) is activated during the limited energy available to the cells. AMPK triggers the ULK1 complex, which activates Vps34 and Beclin-1, leading to the constitution of the autophagosome [48]. Moreover, AMPK stimulates the elongation stage, followed by the function of LC3 and ATG5-ATG12 [49]. During the fusion of the autophagosome with a lysosome in the final stage, several proteins, including synaptosome-associated protein 29 (SNAP29), Syntaxin 17 (STX17), vesicle-associated membrane protein 8 (VAMP8), homotypic fusion and protein sorting-tethering complex (HOPS), LC3/gamma-aminobutyric acid receptor-associated protein (GABARAP), and Rab7, are involved [50, 51]. In the energy-deprived state, inhibition of mitogen-activated protein kinase (MAPK) results in inhibition of mTOR phosphorylation and hence activation of autophagy [52, 53].

## 3. Statins

**Figure 1:**
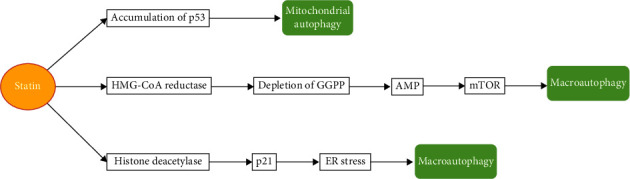
Mechanisms by which statins promote autophagy.

Statins are plant-derived chemicals that inhibit the HMG-CoA, therefore lowering the levels of LDL [54–56]. By inhibition of HMG-CoA, statins interfere with the mevalonate (MVA) pathway or the HMG-CoA reductase pathway, leading to reduced generation of LDL cholesterol and development of autophagy (Figure 1) [57, 58]. The first statins identified were mevastatin (mevinolin) extracted from *Penicillium citrinum* [59]. Next, lovastatin was isolated from *Aspergillus terreus* and got approval by the Food and Drug Administration in 1987 [60]. That notwithstanding, due to adverse effects during trials, cerivastatin was banned. Subsequently, several compounds of the statin family were identified, including pravastatin, simvastatin, fluvastatin, and atorvastatin.

A novel functional pathway of statin had recently been described by Schonewille et al. According to their investigations, statins could promote the excretion of cholesterol through the fecal route [61]. Nonetheless, statins probably act through perturbation of the MVA pathway and reduce the serum cholesterol level [62]. Both farnesyl diphosphate (FPP) and geranylgeranyl diphosphate (GGPP), which are generated through the MVA pathway, are critical in the proper functioning of GTPase proteins, such as Ras, Rac, and Rho [62–65]. By regulating such GTPase proteins, statins may affect cancer cells and confer a therapeutic pathway for treating various malignancies [66]. Additionally, statins lower the LDL-C level, which correlates with a reduced risk of CVD [67, 68]. Over the past few decades, the prescription of statins has been increased globally and will continue to do so in the upcoming decades [69]. Consequently, further trials need to be implemented to characterize the safety and adverse effects of statins.

The potential impact of statins in reducing diabetes mellitus (DM) has also been evaluated. According to the observations, long-term or first-time use of statins was not associated with DM incidence [70]. Nonetheless, a recent study on short-term consumption of statins (in comparison to those that have never used statins) showed that it might promote susceptibility to the development of DM [71]. Hence, the rate of DM might increase after the widespread use of statin, which requires attention. Despite several adverse effects, statins effectively lower lipid and treatment of CVD according to the outcomes by the meta-analysis of clinical trials [72, 73]. The beneficial characteristics of statins are not confined to lowering lipids and improvement of dyslipidemia. Statins also have several properties that potentiate them in the respective therapy of different disorders, including anti-inflammatory [74, 75], antioxidant [76, 77], neuroprotective [78, 79], and antitumor [80]. Consumption of statins has also been related to a decreased rate of Alzheimer's disease and the development of dementia [81]. Additionally, the long-time use of statins has been shown to promote the survival of cancer patients, suggesting the potential of statins in the treatment of tumors [82].

One of the limiting points of statins is their low bioavailability. For example, simvastatin has the lowest bioavailability of 5%, and pravastatin, rosuvastatin, and atorvastatin have bioavailability ranging from 10 to 20% [83]. That notwithstanding, attempts have been developed to promote the bioavailability of statins. As an example, nanocarriers have been tried to increase the bioavailability of statins [84].

## 4. Antitumor Effects of Statins

Despite an increase in the incidence of malignancies, the mortality rate has been reduced because of progression in the development of therapeutic strategies over the past few years [85]. Regarding the critical role of autophagy in the pathogenesis of cancers, modulation of autophagy might be a promising therapeutic approach. Studies have revealed that simvastatin stimulates the ERK1/2 and Akt pathways and therefore dysregulates autophagy flux and promotes cancer cell death [86]. Lovastatin has been used as a complementary agent in the treatment of malignancies. For example, lovastatin has been used as an adjutant in cisplatin-based therapy for the treatment of cancers. Lovastatin therapy promotes the levels of LC3B-II, a marker for autophagosomes, leading to upmodulation in the autophagic cell death and reduced viability of the tumor cells in malignant pleural mesothelioma [87]. Through triggering the autophagy pathway via Rac/phospholipase C/inositol 1,4,5-triphosphate axis, it was seen that lovastatin significantly reduced the migration and survival of the human malignant peripheral nerve sheath tumor cell lines ST88-14, STS-26T, and NF90-8 [88]. Stimulation of autophagic cell death excels the apoptotic cell death during tumor therapy. This is because of the resistance of cancer cells to death by apoptosis and the stimulatory properties of the apoptosis-bearing tumor cells on the proliferation and vitality of the cells in the vicinity. Additionally, the combination treatment of tumor cells with farnesyltransferase inhibitor and lovastatin resulted in efficient autophagy of the tumor cells [88]. It was shown that simvastatin suppressed the nuclear factor-*κ*B (NF-*κ*B) in the human gastric cancer cells and decreased the proliferation of these cells. Furthermore, simvastatin increased the sensitivity of gastric tumor cells to the chemotherapy compound capecitabine [89]. Hence, statins might indirectly confer antitumor effects by increasing chemosensitivity in the tumor cells and increasing chemotherapy's efficacy. Therefore, statins can be used in combination with chemotherapy to obtain better outcomes.

During the progression of tumor cells, angiogenesis plays a critical role. Several therapeutic strategies have been developed to inhibit angiogenesis pathways to suppress tumor development [90, 91]. It was shown that atorvastatin promoted autophagy in human umbilical vein endothelial cells and inhibited angiogenesis. Additionally, atorvastatin promoted the expression of LC3II and decreased the survival and proliferation of malignant cells [92]. Furthermore, pitavastatin induced autophagy in the melanoma cells by promoting the LC3II levels [93]. Rosuvastatin was shown to stimulate autophagy in papillary thyroid carcinoma. It was demonstrated that rosuvastatin decreased cell viability and caused a G1 phase arrest in the papillary thyroid cell line. Hence, rosuvastatin may be an alternative treatment for refractory papillary thyroid cancer [94]. Studies support the antitumor effect of fluvastatin by promoting autophagy. It was suggested that fluvastatin developed autophagy by upmodulation of the expression of the autophagy-related gene (ATG) 5 and ATG7 and therefore repressed the metastasis of lung adenocarcinoma to bone tissue [95]. Additionally, fluvastatin increased LC3II levels and induced autophagy, reducing proliferation and survival of lymphoma cells *in vitro* [95]. Therefore, the antitumor effects of statins may be due to suppressing the angiogenesis in the tumor tissue, above and beyond the direct effect on the tumor cells themselves.

## 5. Osteoarthritis Therapy

Osteoarthritis (OA) is defined as a bone and joint disorder characterized by progressive degradation of cartilage [96]. The role of autophagy in the modulation of chondrocytes and etiopathogenesis of OA has been demonstrated [80]. On the other side, reports show the beneficial therapeutic characteristics of statins (like simvastatin) in patients with OA. However, an inflammatory state might suppress autophagy pathways and exacerbate the OA [97]. It was observed that simvastatin resulted in the downregulation of matrix metalloproteinases (MMPs) and promoted autophagy in OA patients. Simvastatin developed the autophagy pathway by suppressing the phosphorylation of mTOR and upmodulating LC3 [97]. It has been shown that pravastatin also modulates autophagy in rat articular chondrocytes [98] and ameliorates OA patients. As a promising therapeutic strategy, the administration of pravastatin in patients with OA might promote autophagy, which results in the deceleration of cartilage degradation. It was demonstrated that pravastatin therapy in the endothelial progenitor cells led to MAPK signaling inhibition, which in turn resulted in activation of autophagy through promoting the transcription of expression of Beclin-1, LC3II, ATG7, and ATG12. Pravastatin also inhibited mTOR and activated MAPK signaling, leading to the induction of autophagy and thereby causing improvement in the vascular necrosis in the femoral head [99]. Recently, a meta-analysis by Wang et al. indicated that statin administration might not be associated with a lower risk of incidence and progression of OA. Nonetheless, an opposite function of atorvastatin and rosuvastatin was observed in OA [100]. Hence, the potential beneficial effect of statins on OA improvement is a matter of debate, but it might underlie the suppression of autophagy in the chondrocytes by several statins.

## 6. Neuroprotective Effects of Statins

Among the significant neurodegenerative disorders with a considerable rate of mortality are Parkinson's disease (PD), multiple sclerosis (MS), amyotrophic lateral sclerosis (ALS), and AD. Evidence has associated the etiopathogenesis of neurodegenerative disorders to aging as well as impairments in the autophagy pathways [101, 102]. In patients with ALS, there is a motor neuron death, and simvastatin has been shown to suppress the GGPP synthesis, develop autophagic vacuoles, and decrease LC3II/I levels, which results in reduced autophagy in the neurons and ameliorates disease manifestations [103]. Rosuvastatin was shown to provide therapeutic neuroprotective effects in cerebral ischemic/reperfusion injury [104]. Moreover, it was revealed that the combination treatment of rats (adult male Sprague-Dawley rats receiving middle cerebral artery occlusion surgery as an animal model of cerebral ischemia/reperfusion injury) by rosuvastatin and resveratrol could efficiently reduce the activation of caspase-3 and serum levels of interleukin- (IL-) 1*β*. Besides, it promoted the expression of Beclin-1 and LC3II and increased the ratios of Bcl-2/Bax and LC3II/LC3I, leading to autophagy perturbation. These events reduced the cerebral infarct volume and the neurologic defective score, leading to ameliorating disease outcomes [104]. *In vitro* investigations have demonstrated the neuroprotective effects of rosuvastatin. It was shown that rosuvastatin decreased the expression of *α*-synuclein and mTOR but increased the expression of Beclin-1 and AMPK pathways in the rotenone-induced neurotoxicity model in SH-SY5Y cells (an *in vitro* model of PD), leading to activation of autophagy [105]. Neuroinflammation-mediated neurodegeneration was alleviated through a combination of statins, including pravastatin, fluvastatin, simvastatin, pitavastatin, atorvastatin, and rosuvastatin [106]. Consequently, statins may modulate the autophagy pathways in the neurons and promote neuroprotective impressions in neurodegenerative diseases.

## 7. Cardioprotective Effects of Statins

Modulation of autophagy has been associated with cardioprotective effects in CVD [107]. Studies on the effects of atorvastatin on the mouse model of vulnerable atherosclerotic plaque indicated that atorvastatin could promote the stability of vulnerable atherosclerotic plaques and reduced the lesions in the aorta. This was related to decreased lipid deposition as well as a reduced inflammatory state which was attributed to the inhibition of activation of nucleotide-binding oligomerization domain-like receptor pyrin domain containing 3 (NLRP3) inflammasome and decreased levels of inflammatory cytokines, such as tumor necrosis factor- (TNF-) *α*, IL-1*β*, and IL-18. Furthermore, atorvastatin promoted the autophagy pathway in the cardiomyocyte by an mTOR-dependent approach [108]. Besides, pitavastatin prevented the autophagy pathway by downmodulating Beclin-1, resulting in a diminished cardiomyocyte injury [109]. Additionally, rosuvastatin was also associated with a cardioprotective activity that developed the autophagy pathway by promoting Beclin-1 expression and MAPK activation through inhibition of mTOR signaling [110]. Inhibition of AMPK to prevent autophagy in the cardiomyocytes resulted in abrogation of the cardioprotective function of rosuvastatin. Additionally, simvastatin was shown to suppressed autophagy signaling via reducing the LC3II/LC3I ratio and downregulating P62 expression and therefore conferred a cardioprotective effect [111]. This evidence implies the modulation of the autophagy pathways in the cardiomyocytes by statins, leading to protection against cardiac injuries.

## 8. Challenges of Statin Therapy

Although several pros have been associated with statin therapy, some cons still exist. For instance, the bioavailability and the circulation of lipophilic statins upon oral intake are relatively low because of the first-pass metabolism by the liver and removal through the digestive process. The lower oral bioavailability may stem from weak solubility as well as low permeability. Pharmaceutical studies have been developing new strategies to overcome the lower oral bioavailability of statins [112]. Other than bioavailability, data have reported that statins may cause myopathies through different proposed mechanisms [113–115]. This effect might be self-limited myotoxicity through the direct effect of statins on the muscle and autoimmune responses by autoantibodies against 3-hydroxy-3-methylglutaryl coenzyme A reductase (HMGCR) [116]. Additionally, clinical trials have indicated that statin therapy might increase the risk of developing diabetes mellitus in prediabetic individuals. Approximately 10-20% out of 10,000 subjects receiving statin might develop diabetes annually [117]. As the rate of hemorrhagic stroke is increased when the cholesterol levels are reduced in the blood, patients with statin therapy may exercise caution [118]. Even though statin therapy has been associated with increased liver enzymes, hepatotoxicity might be rarely seen [119]. Regarding the disadvantages of statin therapy (even if rare), patients with specific considerations should be evaluated before initiating the treatment regimen.

## 9. Conclusions and Future Directions

A bulk of investigations have indicated the ameliorative effects of statins on treating a wide range of disorders (Table 1). The therapeutic properties of statins have been attributed to the modulation of various molecules involved in the autophagy pathways. Autophagy is a mechanism of the cells under stress to promote survival and the resolution of the damaged and superfluous organelles within the cells. Considering the vital role of autophagy in maintaining healthy conditions and the importance of autophagy dysregulation in the etiopathogenesis of several disorders, modulation of the autophagy signaling pathway might confer a promising therapeutic approach. As a well-known statin, simvastatin has been associated with cardioprotective and neuroprotective properties. Furthermore, simvastatin therapy reduces the cancerous behaviors of tumor cells and has been shown to decelerate the progression of OA. Another statin, lovastatin, promotes autophagy and inhibits the proliferation and migration of tumor cells. Additionally, induction of autophagy has been attributed to pitavastatin therapy that reduces the survival of melanoma tumor cells. However, it has been associated with the suppression of autophagy and cardioprotective characteristics. Another statin, fluvastatin, has been shown to induce autophagy and hence exert antitumor properties. Taken together, it seems that modulation of autophagy by statins has promising potential in the protection of neurons and myocytes as well as in decreasing adverse cancerous behaviors of tumor cells and degradation of cartilages in OA patients. That notwithstanding, further experimental and clinical trials are needed to clarify the therapeutic characteristics of statins and safety and toxicity issues.

## Figures and Tables

**Table 1:** Modulation of autophagy by statins in different disorders.

Disease type	Statin	Target cell/disease	Consequence	Reference
Cancer	Simvastatin	Motoneuron-like NSC34 cells, a model of neuroblastoma	Reduction of autophagic vacuoles and LC3II/I decreased cell survival	[103]
	Simvastatin	Breast cancer cells of MDA-MB-231	Inhibition of autophagy Reduced survival of breast cancer cells	[86]
	Lovastatin	Malignant mesothelioma ZL55 cancer cells	Stimulation of autophagy Reduced survival of malignant mesothelioma cells	[87]
	Lovastatin	Human malignant pleural mesothelioma ACC-MESO1 cells	Induction of autophagy Decreased survival of malignant pleural mesothelioma	[120]
	Lovastatin	Human malignant peripheral nerve sheath tumor ST88-14 and NF90-8 cells	Perturbation of autophagy flux, induction of nonapoptotic cell death Decreased survival of malignant peripheral nerve sheath tumor cells	[88]
	Atorvastatin	Breast cancer MDA-MB-231 cells	Stimulation of autophagy Inhibition of breast cancer cell proliferation	[92]
	Pitavastatin	Human melanoma WM115 and A375 cells	Induction of autophagy Decreased survival of melanoma cells	[93]
	Rosuvastatin	Human papillary thyroid cancer cells	Suppression of autophagy Induction of apoptosis in papillary thyroid cancer cells	[94]

Osteoarthritis	Simvastatin	Mouse model of OA	Stimulation of autophagy Deceleration of OA progression	[97]

Cardiovascular diseases	Pitavastatin	Cardiomyocyte	Suppression of autophagy Decreased cardiomyocyte injury	[109]
	Simvastatin	Male Sprague-Dawley rat	Suppression of autophagy Decreased cardiomyocyte injury	[111]
	Atorvastatin	Mouse model of atherosclerosis	Stimulation of autophagy Suppression of inflammation Increased stability of vulnerable atherosclerotic plaques	[108]
	Rosuvastatin	Myocardial infarction model rat	Induction of autophagy Improved acute myocardial infarction	[110]

Neurodegenerative diseases	Rosuvastatin	Rotenone-induced neurotoxicity model, PD SH-SY5Y cells	Induction of autophagy Neuroprotective function against PD	[105]
	Atorvastatin, pitavastatin, fluvastatin, pravastatin, simvastatin, and rosuvastatin	*In vitro* model of lipopolysaccharide- (LPS-) induced neuroinflammation	Suppression of autophagy Neuroprotective function	[106]

## Data Availability

There is no raw data associated with this review.
